# The effectiveness of Question Prompt Lists (QPL) in enhancing treatment outcomes for cancer patients among tribal population in Meghalaya: A quasi-experimental design

**DOI:** 10.1371/journal.pone.0338482

**Published:** 2025-12-22

**Authors:** Redolen Rose Dhar, Bhageerathy Reshmi, Ramesh Holla, Vennila Jaganathan, Kripa Josten, Anisha Mawlong

**Affiliations:** 1 Department of Health Information Management, Manipal College of Health Professions (MCHP), Manipal Academy of Higher Education (MAHE), Manipal, Karnataka, India; 2 Department of Community Medicine, Kasturba Medical College, Mangalore, Manipal Academy of Higher Education (MAHE), Mangalore, Karnataka, India; 3 Statistics, Manipal College of Health Professions (MCHP), Manipal Academy of Higher Education (MAHE), Manipal, Karnataka, India; 4 Department of Radiation Oncology, Civil Hospital Shillong, Laban, Meghalaya, India; University of Birmingham, UNITED KINGDOM OF GREAT BRITAIN AND NORTHERN IRELAND

## Abstract

**Background:**

Cancer remains a leading cause of death worldwide, with a significant burden in India’s northeastern states, including Meghalaya. Patients from tribal communities often face unique sociocultural and communication barriers that affect their engagement in care. Effective patient–physician communication improves satisfaction, adherence, and quality of life. This study aims to assess the consultation satisfaction and effectiveness of a Question Prompt List (QPL) in enhancing patient–physician communication and promoting informed decision-making among cancer patients in a tribal population in Meghalaya, India.

**Methods:**

A quasi-experimental design was employed involving 60 participants diagnosed with head and neck, gastrointestinal, or respiratory cancers. Participants were assigned to an intervention group that received the QPL and a control group that received standard care. Data on consultation satisfaction and patient feedback were collected across two follow-up sessions.

**Results:**

The intervention group demonstrated statistically significant improvements in multiple domains of consultation satisfaction, including physicians’ responsiveness, patients’ confidence in asking questions, understanding of disease and treatment plans, and discussions of financial aspects. In contrast, the control group showed no significant change across follow-ups. Patient feedback indicated high levels of agreement regarding the QPL’s usefulness, clarity, and ability to facilitate meaningful communication.

**Conclusion:**

The QPL significantly enhanced patient satisfaction and engagement, demonstrating its effectiveness as a low-cost, culturally adaptable communication tool for oncology consultations among tribal populations. Integrating QPLs into routine oncology practice could strengthen patient-centred care and reduce communication barriers in underserved regions.

## Background

Cancer is the leading cause of death worldwide [1]. The World Health Organisation (WHO) projects a 45% increase in global cancer deaths from 2008 to 2030 [2], with the mortality burden expected to be significantly higher in low-income countries compared to developed economies [3]. Cancer not only significantly impacts patients’ health but also places a substantial economic strain on families, particularly affecting younger individuals with lower incomes more severely [4]. Those from less affluent backgrounds often choose more basic treatments, which may have lower survival rates and higher toxicity due to cost considerations [5]. Additionally, financial constraints lead to non-adherence in 45% of patients [6], and those facing financial issues are more likely to delay or skip treatments altogether [7]. Decision-making in cancer treatment is complex, even without considering the cost. Patients, balancing fear and hope, strive to make a choice that optimises survival while weighing medical benefits and risks [8]. However, cost-related concerns often hinder open communication. Many patients feel uncomfortable raising financial issues with their doctors, perceiving it as inappropriate or fearing embarrassment. Likewise, physicians often cite a lack of time or resources as reasons for not addressing these discussions [9]. This communication gap contributes to patient distress and poor treatment adherence [10–12]. Effective communication is crucial for establishing a robust patient-physician relationship in cancer care [13–16]. It enhances patient satisfaction, reduces distress, promotes faster recovery, and improves pain control, adherence to treatment, and quality of life [17–19]. Conversely, communication breakdowns increase stress, decrease job satisfaction, and contribute to burnout [19]. Unsatisfactory consultations can lead to patient dissatisfaction and, in extreme cases, may result in misunderstandings and even litigation [20]. Thus, improving communication benefits both patients and healthcare providers [19].

QPLs are structured, evidence-based lists of questions designed to help patients initiate and guide conversations with healthcare providers during consultations [21]. QPLs empower patients to seek clarifications, express concerns, and participate more actively in decision-making. By facilitating dialogue, they help ensure that consultations are patient-centred, responsive to individual needs, and inclusive of patients’ preferences [22,23]. QPLs have been effectively utilised in diverse healthcare contexts, including oncology, palliative care, and chronic disease management [24]. Evidence shows that their use enhances patient participation, improves information recall, and reduces pre-consultation anxiety [25,26]. In oncology settings, QPLs have been shown to be particularly valuable in promoting open discussions about diagnosis, treatment options, side effects, and prognosis, thereby enhancing shared decision-making [22]. However, despite these demonstrated benefits, limited evidence exists regarding their use and effectiveness in low-resource or culturally distinct settings such as India.

In India, cancer incidence varies considerably by region, with the Northeastern states reporting some of the highest rates nationwide. According to the Population-Based Cancer Registry [27]. Meghalaya ranks among the top states for cancer incidence, with Oesophageal cancer being the leading site of cancer in both genders (AAR of 75.4 in men and 33.6 in women) [28,29]. Limited oncology infrastructure, late-stage diagnosis, and substantial out-of-pocket expenses further exacerbate the burden for patients and their families. The majority of Meghalaya’s population belongs to tribal communities, where health-seeking behaviours and communication patterns are shaped by distinct socio-cultural beliefs, traditional knowledge systems, and linguistic diversity [28,29]. These factors often hinder effective dialogue between patients and healthcare providers, leading to lower patient participation in decision-making. Testing a QPL in this context is essential to determine its feasibility, cultural acceptability, and potential impact on improving patient–physician communication and satisfaction. Therefore, this study aims to assess the consultation satisfaction and effectiveness of a Question Prompt List (QPL) in enhancing patient–physician communication and promoting informed decision-making among cancer patients in a tribal population in Meghalaya, India.

## Methods

### Study design

The study was conducted at the Civil Hospital, Shillong, located in the East Khasi Hills District of Meghalaya, India, between September 1, 2023, to February 22, 2024. Civil Hospital is one of the largest public-sector hospitals in Meghalaya, recognised for providing comprehensive cancer care services. A quasi-experimental design was adopted, with participants divided into an intervention group and a control group. The quasi-experimental approach was chosen because it allows for evaluating the effectiveness of an intervention (the QPL) in a real-world clinical setting, where random assignment is not feasible due to ethical and logistical constraints. This design provides flexibility in studying behavioural and communication-related interventions while maintaining comparability between groups.

The control group received standard oncology care, consisting of routine consultations without the use of additional communication tools or materials. The intervention group received a QPL prior to the second consultation after diagnosis, allowing sufficient time for patients to process their diagnosis and initial treatment discussions, while enabling an assessment of the QPL’s impact during follow-up consultations. Typically, the second consultation occurred within 2–3 weeks after the initial diagnostic consultation, coinciding with treatment planning discussions. A total of 60 participants were recruited for the study, meeting specific inclusion criteria. These criteria included having a diagnosis of head and neck, gastrointestinal, or respiratory cancers, receiving treatment at the hospital, and the ability to communicate and provide informed consent. Informed consent was obtained from all participants prior to their inclusion in the study.

### Ethical consideration

The study received ethical approval from two institutional review boards: the Kasturba Hospital Institutional Ethics Committee (IEC1:16/2023) and the Institutional Ethics Committee of the Pasteur Institute (IEPCI ID: DHSR/IECP101/23/01). Prior to participation, all participants provided written informed consent, and the process was documented and securely stored by the research team. The study adhered to the principles outlined in the Declaration of Helsinki. The study was registered with the Clinical Trials Registry India (CTRI/2023/08/056011; http://ctri.nic.in/) on August 2nd, 2023 (REF/2023/05/067336).

### Question prompt list

QPL is a structured communication tool designed to help patients prepare questions for healthcare providers, promoting active participation and reducing disparities in patient-physician communication [30]. QPLs typically consist of concise evidence-based questions related to disease management, treatment options, prognosis and psychosocial or financial concerns [31]. Previous research has demonstrated that QPLs can enhance patient engagement, improve satisfaction and reduce anxiety during medical consultations [32–34]. By encouraging patients to express concerns and seek clarifications, QPLs serve as a low-resource, high-impact communication strategy in oncology care [35].

### Design of Question Prompt List

The development of the Question Prompt List (QPL) followed an iterative, multi-phase process that incorporated evidence from the literature, expert input, and cultural validation to ensure contextual relevance and accuracy. The initial draft was developed through a comprehensive review of existing QPLs and communication aids used in oncology and other clinical settings [22,30–32,35–49]. Draft questions were then refined with input from oncologists, psychologists, and public health experts from Civil Hospital Shillong and Kasturba Medical College (KMC), Manipal. Feedback was also sought from nursing faculty and health communication specialists to ensure the QPL aligned with clinical workflow and matched the literacy levels of patients. The draft QPL was organised into ten thematic domains, comprising 85 questions that cover areas such as cancer diagnosis, treatment plans, side effects, treatment costs, insurance coverage, out-of-pocket expenses, and treatment scheduling, with additional space provided for patients to add their own questions. The finalised draft was translated into the local Khasi language and back-translated to ensure linguistic accuracy and comprehension (Annexure 1). To validate the tool culturally, a pilot review was conducted with a small group of patients (n = 5) and caregivers to assess clarity, cultural appropriateness, and relevance. Minor linguistic modifications were made based on their feedback, following which the expert panel approved the final version of the QPL for implementation.

### Sample size

The sample size was determined using the formula for comparing two independent means, with 80% power and a 95% confidence level [50]. The estimates were based on findings from previous studies [51] where the mean satisfaction scores for the control and intervention groups were 6.5 ± 2.4 and 4.3 ± 2.5, respectively, yielding an effect size of 0.82. Accounting for a 20% non-response rate, the final sample size was set at 30 participants per group (total = 60).

### Participants’ recruitment and assignment

A total of 60 participants were enrolled in the study, with 30 assigned to the control group and 30 to the intervention group. Recruitment was conducted in the oncology department of Civil Hospital, Shillong, over a six-month period. Eligible participants were adults aged between 30 and 65 years who had been diagnosed with head and neck, gastrointestinal, or respiratory cancers (Stages I–IV), were attending their second consultation after diagnosis, were proficient in either Khasi or English, and had provided informed consent. Patients who were critically ill, receiving palliative care, or unable to participate in follow-up interviews were excluded from the study. To minimise potential bias, recruitment for the control group was conducted first over a three-month period, followed by recruitment for the intervention group during the subsequent three months. This sequential approach ensured that both groups were drawn from comparable populations while preventing contamination between participants in the two groups.

### Study procedure

**Fig 1:**
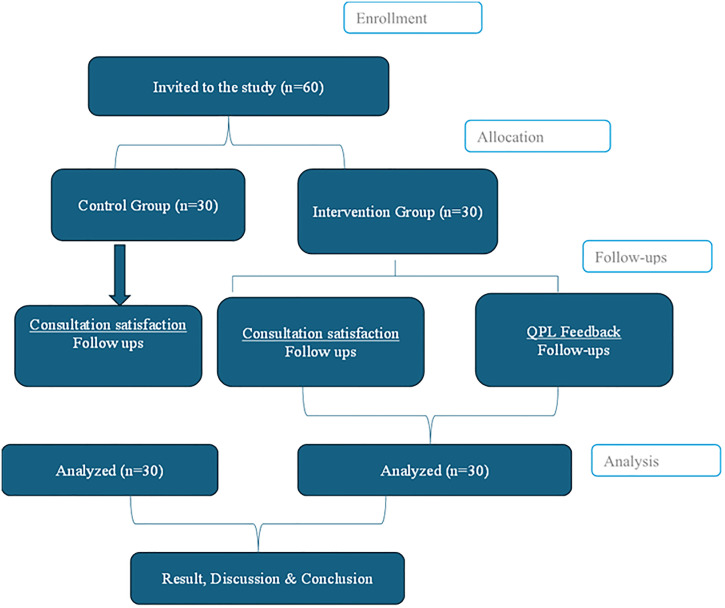
CONSORT flow diagram of participant recruitment, allocation, follow-ups, and analysis.

As illustrated below, the CONSORT flow diagram of participant recruitment, allocation, follow-up, and analysis [52] (Fig 1). The study employed a sequential recruitment process to allocate participants into the intervention and control groups. To minimise potential bias, recruitment for the control group was conducted three months prior to the enrolment of the intervention group. Participants were recruited daily based on predefined eligibility criteria. Eligible patients were informed about the study and invited to participate upon identification. Those who provided informed consent were enrolled and completed baseline socio-demographic data collection. Participants in the control group received standard oncological care without any additional intervention. Their consultations followed the routine clinical approach without providing the Question Prompt List (QPL) or any structured communication tools. Consultation satisfaction for the control group was assessed at two follow-up points: the first follow-up during the second visit and the second follow-up during the next clinical visit, with data collected after each consultation. Following the completion of data collection for the control group at the end of the three months, recruitment for the intervention group commenced. Participants in the intervention group received a Question Prompt List (QPL) before their second consultation with an oncologist. The QPL facilitated patient engagement by enabling participants to review and prepare relevant questions regarding their diagnosis, treatment options, financial considerations, and overall care. For the intervention group, consultation satisfaction was similarly assessed at two follow-up points: the first follow-up during the second visit and the second follow-up during the next visit. Participants also gave feedback that was specific to the QPL at both follow-up points after each consultation. This structured approach ensured a systematic evaluation of the QPL’s effectiveness in enhancing patient engagement and improving patient satisfaction with consultations among patients with cancer.

### Measures

The primary outcome of this study was to assess patient satisfaction with consultations among those with cancer in both the intervention and control groups. Consultation satisfaction was evaluated using an 8-item Likert scale adapted from Roter [51] and Freemon et al. [53] based on previous research [54], which ranged from 1 (“very dissatisfied”) to 5 (“very satisfied”) [55]. The scale measured patient-centred aspects, including physician responsiveness, patient ability to ask questions, understanding of the disease and treatment plan, discussion of treatment costs, and overall consultation satisfaction. Additionally, the effectiveness of the Question Prompt List (QPL) in enhancing patient engagement and communication was assessed using a 14-item Likert scale-based questionnaire [22,30,42,54], adapted from prior studies [55]. This scale measured patients’ perceptions regarding the QPL’s usefulness, ease of understanding, relevance, and ability to facilitate communication with their physician [42,43,45,47,54]. Comparisons of consultation satisfaction scores were conducted between the intervention and control groups at two follow-up intervals to determine the impact of the QPL. The intervention group’s satisfaction levels were analysed across follow-ups to assess changes over time. Similarly, patient feedback on the QPL was analysed to evaluate its effectiveness in fostering meaningful patient-physician interactions.

### Data quality assurance and missing data handling

All collected data were cross-verified daily by the principal investigator for completeness and accuracy. Double data entry was performed to reduce transcription errors. Missing data were not observed as there were no dropouts during the study period.

### Statistical analysis

Data analysis was conducted using JAMOVI (Version 2.3) and JASP (Version 0.19.1.0). We carefully reviewed the data collected from the patients and reported descriptive statistics for the socio-demographic variables. To assess the association between the variables, we utilised the Chi-Square test or McNemar’s test, employing Fisher’s exact test whenever the expected cell count was less than 5. Statistical significance was established with a p-value of less than 0.05. There were no dropouts or follow-ups during data collection, resulting in no missing data. The Chi-Square test was used to examine the association between categorical variables across independent groups (e.g., control vs. intervention). This non-parametric test is suitable for data presented as frequencies or counts, provided that the expected cell frequencies are sufficiently large (generally ≥5 in most cells). When one or more expected cell frequencies fell below 5, Fisher’s exact test was applied instead of the Chi-Square test. Additionally, the McNemar test was employed to assess changes in paired categorical responses before and after the intervention within the same group.

## Results

### Baseline characteristics

Table 1 outlines the key characteristics of the study participants. The majority of participants in both groups were male and married. There was a slightly higher proportion of participants aged 41–50 years in the control group (36.7%) compared to the intervention group (30.0%). In contrast, the intervention group had a higher percentage of participants aged 51–60 (40.0%) than the control group (20.0%). Most participants resided in rural areas, with a slightly higher proportion in the control group (90.0%) compared to the intervention group (83.3%). The majority of participants in both groups belonged to the lower or upper-lower socioeconomic classes (63.3% and 73.3%, respectively), indicating that financial constraints may have influenced their healthcare-seeking patterns. Most participants were Christians, reflecting the region’s predominant Christian demographic. Chewing tobacco and betel nut were common among participants, highlighting the prevalence of lifestyle-related risk behaviours in this population. Clinically, gastrointestinal cancers were more frequent in the intervention group (56.7%), whereas head and neck cancers were predominant in the control group (50%). Most participants were diagnosed at advanced stages (Stage II or III); however, stage details were unavailable for some cases due to incomplete medical records. The baseline comparability between the control and intervention groups strengthens the validity of the subsequent between-group analyses. Moreover, the predominance of rural and low-income participants underscores the importance of patient-centred interventions such as the Question Prompt List (QPL), which can enhance communication, engagement, and understanding among socioeconomically disadvantaged populations.

**Table 1:** Baseline characteristics of the study participants.

Characteristic	Categories	Control (N = 30)	Intervention (N = 30)
		N (%)	N (%)
Gender	Female	7 (23.3)	12 (40.0)
	Male	23 (76.7)	18 (60.0)
Marital Status	Married	30 (100.0)	30 (100)
Age group	30-40	3 (10.0)	3 (10.0)
	41-50	11 (36.7)	9 (30.0)
	51-60	6 (20.0)	12 (40.0)
	>61	10 (33.3)	6 (20.0)
Religion	Christian	28 (93.3)	27 (90.0)
	Non-Christian	2 (6.7)	3 (10.0)
Residence	Rural	27 (90.0)	25 (83.3)
	Urban	3 (10.0)	5 (16.7)
Type of family	Joint	0 (0)	7 (23.3)
	Nuclear	30 (100.0)	23 (76.7)
Family Size	Less than 3	2 (6.7)	1 (3.3)
	More than 4	28 (93.3)	29 (96.7)
Socio-Economic Class	Lower (V)	10 (33.3)	6 (20.0)
	Lower middle (III)	1 (3.3)	2 (6.7)
	Upper Lower (IV)	19 (63.3)	22 (73.3)
Substance used	Chewing beetle nuts, tobacco	8 (26.6)	13 (43.3)
	Smoker, alcoholic, chewing tobacco	22 (73.3)	17 (56.7)
Types of Insurance	MHIS	27 (90.0)	26 (86.7)
	NIL	3 (10.0)	4 (13.3)
Type of cancer	Digestive/Gastrointestinal	11 (36.7)	17 (56.7)
	Head and Neck	15 (50.0)	11 (36.7)
	Respiratory/Thoracic	4 (13.3)	2 (6.7)
Stage of Cancer	Not Disclosed	28 (93.3)	26 (86.7)
	Stage 2	1 (3.3)	2 (6.7)
	Stage 3	1 (3.3)	2 (6.7)

### Association between control and intervention (first follow-up)

As shown in Table 2, all items except patient satisfaction with understanding the condition of the disease demonstrated statistically significant differences (p < 0.05) between the control and intervention groups at the first follow-up. Patients in the intervention group reported markedly higher satisfaction in domains such as physicians’ responses to questions, discussion of treatment costs, comprehension of treatment plans, and overall consultation satisfaction. These results suggest that exposure to the QPL before the second consultation increased patient engagement and confidence in interacting with physicians. The lack of a significant difference for “understanding of disease condition” may reflect that this aspect was addressed uniformly during standard consultations.

**Table 2:** Association between the control and intervention 1^st^ follow-up on the consultation satisfaction.

Items Control 1^st^ follow up	Category	Intervention 1^st^ follow up			
		Satisfied or very satisfied N (%)	Neutral N (%)	Dissatisfied or very dissatisfied N (%)	p-value
Physicians response to patients questions	Satisfied or very satisfied	24 (100)	0 (0)	24 (100)	<.001
	Neutral	0 (0)	5 (100)	0 (0)	
	Dissatisfied or very dissatisfied	0 (0)	0 (0)	1 (100)	
Patient satisfaction on asking questions from the physicians	Satisfied or very satisfied	0 (0)	1 (5)	19 (95)	<.001
	Neutral	10 (100)	0 (0)	0 (0)	
Patients satisfaction on understanding the condition of the disease	Satisfied or very satisfied	17 (100)	1 (0)	0 (0)	0.816
	Neutral	0 (0)	10 (100)	0 (0)	
	Dissatisfied or very dissatisfied	0 (0)	0 (0)	3 (0)	
	Neutral	0 (0)	5 (100)	0 (0)	
	Dissatisfied or very dissatisfied	22 (88)	0 (0)	3 (12)	
Patients satisfaction on comprehending the treatment plan	Satisfied or very satisfied	17 (100)	0 (0)	0 (0)	<.001
	Neutral	0 (0)	12 (100)	0 (0)	
	Dissatisfied or very dissatisfied	0 (0)	0 (0)	1 (100)	
Over patients consultation satisfaction	Satisfied or very satisfied	18 (100)	0 (0)	0 (0)	<.001
	Neutral	0 (0)	11 (100)	0 (0)	
	Dissatisfied or very dissatisfied	0 (0)	0 (0)	1 (0)	
Patients satisfaction on physician help understanding with regards to Insurance coverage	Satisfied or very satisfied	21 (100)	0 (0)	0 (0)	<.001
	Neutral	0 (0)	8 (100)	0 (0)	
	Dissatisfied or very dissatisfied	0 (0)	0 (0)	1 (100)	
Patients satisfaction on physician help with out-of-pocket expenses	Satisfied or very satisfied	0 (0)	16 (100)	0 (0)	<.001
	Neutral	8 (100)	0 (0)	0 (0)	
	Dissatisfied or very dissatisfied	1 (16.7)	1 (16.7)	4 (66.6)	

*(chi-square/fishers test was used to find the association between control and intervention 1^*st*^ *follow-up).*

*(All items were rated on a 1–5 scale: 1 is very dissatisfied, and 5 is very satisfied).

### Association between control and intervention (second follow-up)

Table 3 presents the association and satisfaction between the control and intervention groups at the second follow-up. Although improvements were observed in the intervention group, only the domain “patient satisfaction on asking questions” reached statistical significance (p = 0.04). Other domains did not show significant differences (p > 0.05). The sustained improvement in patients’ willingness to ask questions indicates that the QPL continued to empower communication even after initial exposure. However, the absence of statistically significant changes in other domains may be related to the short follow-up duration or limited sample size, suggesting the need for a longer-term evaluation.

**Table 3:** Association between the control and intervention 2^nd^ follow-up on the consultation satisfaction.

Items Control 2^nd^ follow up	Category	Intervention 2^nd^ follow-up			
		Satisfied or very satisfied N (%)	Neutral N (%)	Dissatisfied or very dissatisfied N (%)	p-value
Physicians response to patients questions	Satisfied or very satisfied	22 (75.5)	5 (17.8)	1 (6.7)	0.765
	Neutral	2 (100)	0 (0)	0 (0)	
Patient satisfaction on asking questions from the physicians	Satisfied or very satisfied	20 (76)	0 (0)	6 (24)	0.040
	Neutral	2 (66.6)	1 (33.3)	0 (0)	
	Dissatisfied or very dissatisfied	1 (100)	0 (0)	0 (0)	
Patients satisfaction on understanding the condition of the disease	Satisfied or very satisfied	15 (55.5)	9 (33.3)	3 (11.2)	0.880
	Neutral	1 (50)	1 (50)	0 (0)	
	Dissatisfied or very dissatisfied	1 (100)	0 (0)	0 (0)	
Patients satisfaction on cost discussion and treatment plan	Satisfied or very satisfied	12 (42.8)	9 (32.2)	7 (25)	0.500
	Neutral	0 (0)	1 (100)	0 (0)	
	Dissatisfied or very dissatisfied	1 (100)	0 (0)	0 (0)	
Patients satisfaction on comprehending the treatment plan	Satisfied or very satisfied	13 (50)	3 (11.5)	10 (38.5)	0.473
	Neutral	2 (100)	0 (0)	0 (0)	
	Dissatisfied or very dissatisfied	2 (100)	0 (0)	0 (0)	
Over all patients consultation satisfaction	Satisfied or very satisfied	15 (57.7)	10 (38.5)	1 (3.8)	0.930
	Neutral	2 (66.6)	1 (33.3)	0 (0)	
	Dissatisfied or very dissatisfied	1 (100)	0 (0)	0 (0)	
Patients satisfaction on physician help understanding with regards to Insurance coverage	Satisfied or very satisfied	11 (68.8)	4 (25)	1 (6.2)	0.491
	Neutral	3 (50)	3 (50)	0 (0)	
	Dissatisfied or very dissatisfied	7 (87.5)	1 (12.5)	0 (0)	
Patients satisfaction on physician help with out-of-pocket expenses	Satisfied or very satisfied	2 (25)	5 (62.5)	1 (12.5)	0.472
	Neutral	9 (52.9)	5 (29.4)	3 (17.6)	
	Dissatisfied or very dissatisfied	3 (60)	2 (40)	0 (0)	

*(chi-square/fishers test was used to find the association between the control and intervention 2^*nd*^ *follow-up).*

*(All items were rated on a 1–5 scale: 1 is very dissatisfied, and 5 is very satisfied).

### Within-group association (intervention group: first vs second follow-up)

Table 4 presents the within-group association for the intervention arm. Statistically significant improvements were observed across all satisfaction domains (p < 0.05), including physician responsiveness, discussion of treatment plans and costs, comprehension, and overall satisfaction. These findings suggest that the repeated use of the QPL reinforced patient preparedness, facilitated meaningful discussions, and improved overall consultation satisfaction. The improvement between first and second follow-ups demonstrates that continued exposure to the QPL helped patients internalise questions and communicate concerns more effectively over time.

**Table 4:** Association between the Intervention 1^st^ follow-up and 2^nd^ follow-up on the consultation satisfaction.

Items Intervention 1^st^ follow up	Category	Intervention 2^nd^ follow up			
		Satisfied or very satisfied N (%)	Neutral N (%)	Dissatisfied or very dissatisfied N (%)	p-value
Physicians response to patients questions	Satisfied or very satisfied	24 (100)	0 (0)	0 (0)	<.001
	Neutral	0 (0)	5 (100)	0 (0)	
	Dissatisfied or very dissatisfied	0 (0)	0 (0)	1 (100)	
Patient satisfaction on asking questions from the physicians	Satisfied or very satisfied	19 (100)	0 (0)	0 (0)	<.001
	Neutral	4 (40)	0 (0)	6 (60)	
	Dissatisfied or very dissatisfied	0 (0)	1 (100)	0 (0)	
Patients satisfaction on understanding the condition of the disease	Satisfied or very satisfied	7 (100)	0 (0)	0 (0)	<.001
	Neutral	0 (0)	10 (100)	0 (0)	
	Dissatisfied or very dissatisfied	0 (0)	0 (0)	3 (100)	
Patients satisfaction on cost discussion and treatment plan	Satisfied or very satisfied	13 (59.2)	7 (31.8)	2 (9.0)	<.001
	Neutral	0 (0)	0 (0)	5 (100)	
	Dissatisfied or very dissatisfied	0 (0)	3 (100)	0 (0)	
Patients satisfaction on comprehending the treatment plan	Satisfied or very satisfied	17 (100)	0 (0)	0 (0)	<.001
	Neutral	0 (0)	2 (16.7)	10 (83.3)	
	Dissatisfied or very dissatisfied	0 (0)	1 (100)	0 (0)	
Over all Patients consultation satisfaction	Satisfied or very satisfied	15 (83.3)	1 (5.6)	2 (11.1)	0.008
	Neutral	2 (18.2)	2 (18.2)	7 (63.6)	
	Dissatisfied or very dissatisfied	0 (0)	0 (0)	1 (100)	
Patients satisfaction on physician help understanding with regards to Insurance coverage	Satisfied or very satisfied	21 (100)	0 (0)	0 (0)	<.001
	Neutral	0 (0)	8 (100)	0 (0)	
	Dissatisfied or very dissatisfied	0 (0)	0 (0)	1 (100)	
Patients satisfaction on physician help with out-of-pocket expenses	Satisfied or very satisfied	6 (35.3)	7 (41.1)	4 (23.6)	0.006
	Neutral	8 (88.9)	1 (11.1)	0 (0)	
	Dissatisfied or very dissatisfied	0 (0)	4 (100)	0 (0)	

*(McNemar’s test was used to find the association between the Intervention 1^*st*^ *follow-up and 2*^*nd*^ *follow up).*

*(All items were rated on a 1–5 scale: 1 is very dissatisfied, and 5 is very satisfied).

### Within-group association (control group: first vs second follow-up)

Table 5 shows the associations of the control group across follow-ups. No statistically significant changes were observed in any domain (all p > 0.05). Patient satisfaction in the control group remained relatively stable across consultations, indicating that routine oncological care alone did not enhance patient–physician communication over time. This highlights the importance of structured communication tools, such as QPLs, in enhancing patient engagement.

**Table 5:** Association between the control 1^st^ follow-up and 2^nd^ follow-up on the consultation satisfaction.

Items Control 1^st^ follow up	Category	Control 2^nd^ follow up			
		Dissatisfied or very dissatisfied N (%)	Neutral N (%)	Satisfied or very satisfied N (%)	p-value
Physicians response to patients questions	Satisfied or very satisfied	0 (0)	0 (0)	24 (100)	0.765
	Neutral	0 (0)	5 (100)	0 (0)	
	Dissatisfied or very dissatisfied	1 (100)	0 (0)	0 (0)	
Patient satisfaction on asking Questions from the physicians	Satisfied or very satisfied	16 (80)	3 (15)	1 (5)	0.315
	Neutral	10 (100)	0 (0)	0 (0)	
Patients satisfaction on understanding the condition of the disease	Satisfied or very satisfied	15 (88.2)	1 (5.88)	1 (5.8)	0.880
	Neutral	9 (90)	1 (10)	0 (0)	
	Dissatisfied or very dissatisfied	3 (100)	0 (0)	0 (0)	
	Neutral	5 (100)	0 (0)	0 (0)	
	Dissatisfied or very dissatisfied	23 (92)	1 (4)	1 (4)	
Patients satisfaction on comprehending the treatment plan	Satisfied or very satisfied	13 (76.4)	2 (11.8)	2 (11.8)	0.473
	Neutral	12 (100)	0 (0)	0 (0)	
	Dissatisfied or very dissatisfied	1 (100)	0 (0)	0 (0)	
Over patients consultation satisfaction	Satisfied or very satisfied	15 (83.4)	1 (5.6)	2 (11.1)	0.930
	Neutral	10 (90.9)	0 (0)	1 (9.1)	
	Dissatisfied or very dissatisfied	1 (100)	0 (0)	0 (0)	
Patients satisfaction on physician help understanding with regards to Insurance coverage	Satisfied or very satisfied	11 (52.4)	7 (33.3)	3 (14.3)	0.491
	Neutral	4 (50)	1 (12.5)	3 (37.5)	
	Dissatisfied or very dissatisfied	1 (100)	0 (0)	0 (0)	
Patients satisfaction on pysician help with out-of-pocket expenses	Satisfied or very satisfied	5 (31.2)	2 (12.6)	9 (56.2)	0.374
	Neutral	1 (12.5)	3 (37.5)	4 (50)	
	Dissatisfied or very dissatisfied	2 (33.3)	0 (0)	4 (66.6)	

*(McNemar’s test was used to find the association between the control 1^*st*^ *follow up and 2*^*nd*^ *follow up).*

*(All items were rated on a 1–5 scale: 1 is very dissatisfied, and 5 is very satisfied).

### Patient feedback on QPL

Table 6 summarises participant feedback on the Question Prompt List (QPL). Patients consistently reported high levels of agreement or strong agreement across all items, indicating that the tool was perceived as helpful, easy to understand, and relevant to their needs. Significant improvements were observed in three key areas: the recommendation of the QPL to doctors and patients (p = 0.01), a reduction in anxiety before consultations (p = 0.01), and perceptions of the QPL as being overwhelming to read (p = 0.02). These findings suggest that while the QPL effectively reduced anxiety and enhanced patient confidence during consultations, a small proportion of participants found the tool somewhat lengthy or complex to navigate, underscoring the need for simplified formats and brief pre-consultation orientation to optimise its usability and impact.

**Table 6:** Patient feedback on QPL.

Items	Category	2^nd^ Follow Up		p-value/fisher’s exact
		Agree or Strongly Agree N (%)	Uncertain N (%)	
The QPL Helpful	Agree or Strongly Agree	28 (96.6)	1 (100)	1.00
	Uncertain	1 (3.4)	0 (0)	
The Visit partner found it helpful	Agree or Strongly Agree	26 (89.7)	1 (100)	1.00
	Uncertain	3 (10.3)	0 (0)	
The QPL is easy to understand	Agree or Strongly Agree	28 (96.6)	1 (100)	1.00
	Uncertain	1 (3.4)	0 (0)	
The QPL is relevant and useful	Agree or Strongly Agree	27 (93.1)	1 (100)	1.00
	Uncertain	2 (6.9)	0 (0)	
The QPL helped in asking Questions and concerns	Agree or Strongly Agree	28 (96.6)	1 (100)	1.00
	Uncertain	1 (3.4)	0 (0)	
The QPL helped in putting forth Question and concerns like never before	Agree or Strongly Agree	28 (96.6)	0 (0)	0.67
	Uncertain	1 (3.4)	1 (100)	
The QPL recommends to the Doctors and Patients	Agree or Strongly Agree	26 (92.9)	0 (0)	0.01
	Uncertain	2 (7.1)	2 (100)	
The QPL helps during cost discussion	Agree or Strongly Agree	28 (96.6)	1 (100)	1.00
	Uncertain	1 (3.4)	0 (0)	
The QPL reduce the anxiety	Agree or Strongly Agree	23 (85.2)	0 (0)	0.01
	Uncertain	4 (14.8)	3 (100)	
The QPL is overwhelming to read	Agree or Strongly Agree	26 (96.3)	1 (33.3)	0.02
	Uncertain	1 (3.7)	2 (66.7)	
The QPL helped manage the time	Agree or Strongly Agree	28 (96.6)	1 (100)	1.00
	Uncertain	1 (3.4)	0 (0)	
The QPL helped to be forthcoming during the consultation	Agree or Strongly Agree	28 (96.6)	1 (100)	1.00
	Uncertain	1 (3.4)	0 (0)	
The QPL help me to be confident during the discussion	Agree or Strongly Agree	27 (93.1)	1 (100)	1.00
	Uncertain	2 (6.9)	0 (0)	
I would like to use this QPL for the future consultation	Agree or Strongly Agree	28 (96.6)	1 (100)	1.00
	Uncertain	1 (3.4)	0 (0)	

(* Fisher’s exact test was used to find the association between the 1^*st*^ *follow-up and the 2*^*nd*^ *follow-up of the intervention and the control groups).*

(*All items were rated on a 1–5 scale: 1 is Strongly Disagree, and 5 is Strongly Agree).

## Discussion

This study was conducted in Meghalaya, India, where cancer ranks among the top five leading causes of death due to inadequate healthcare facilities and delays in seeking treatment and diagnosis [56]. Moreover, the state faces challenges in health, education, and poverty, with the poverty rate now at 32.67% [57]. Meghalaya’s largely tribal population faces additional barriers to healthcare access, including geographical isolation, reliance on traditional healing practices, and limited awareness of cancer-care pathways, which are crucial contextual factors when interpreting the effectiveness of interventions such as the Question Prompt List (QPL) [58]. Recent qualitative research from the same region also highlights that cancer patients research in the tribal often experience substantial financial distress and gaps in communication of costs with healthcare providers, underscoring the urgent need for interventions that enhance patient–physician dialogue [59]. Our study demonstrates that using a QPL significantly enhances cancer patients’ satisfaction regarding their oncologist consultations. Comparing the first and second follow-ups, we observed marked improvements in the physician’s ability to answer questions, patients’ confidence in asking them, and understanding of disease conditions, treatment plans, and insurance coverage. These findings align with previous evidence demonstrating that QPLs enhance communication quality and patient engagement in oncology [34,36,60]. Patients who used the QPL reported higher comfort levels in asking questions and greater satisfaction with physician responses compared to the control group. These results align with studies by Bruera et al. and Yeh et al., which have demonstrated that QPLs enhance patients’ understanding and promote structured, meaningful conversations [60,61]. In contrast to findings from high-resource settings, our study suggests that in low-resource, tribal contexts, QPLs play a dual role, bridging informational gaps and fostering trust in healthcare providers. In communities where health decisions are often family-centred and influenced by cultural beliefs, QPLs serve as culturally adaptable frameworks that promote shared decision-making and empower patients to participate actively in their care [34,60].

The control group showed minimal improvement over time, particularly in discussions about treatment costs and plans. This highlights the importance of structured communication tools in bridging gaps in patient–physician interactions. Although satisfaction scores declined slightly during the second follow-up, the overall pattern suggests that sustained physician engagement, reinforcement, and refresher sessions are essential to maintaining QPL benefits [62]. Similar observations have been made in other communication-intervention studies, where initial gains diminished without continued reinforcement [34]. The QPL effectively encouraged active participation and facilitated shared decision-making, consistent with Zetzl et al, who found that QPLs improve interactional empowerment among cancer patients. Previous studies have also shown that QPLs help patients manage anxiety and discuss complex or sensitive issues, including prognosis and end-of-life decisions [34,36]. Our study extends this understanding by incorporating financial concerns such as treatment costs and insurance coverage into the QPL framework, enabling patients to better navigate the economic aspects of their care. While these discussions improved awareness and reduced anxiety, satisfaction related to financial issues was less pronounced than in other domains. This likely reflects physicians’ discomfort with cost communication and limited institutional training to address such discussions [63]. These findings are also consistent with recent qualitative research in the tribal region, which reported that patients often hesitate to raise financial concerns and seek more empathetic, transparent conversations about costs [59]. In the Indian context, Chawak et al. emphasised that culturally sensitive tools, such as QPLs, help overcome hierarchical communication patterns and encourage patient expression, particularly in family-influenced care settings [60,61]. Our findings align with this and suggest that training physicians on the use of QPL, including financial and culturally responsive communication, as well as involving caregivers during consultations, could further enhance its utility. Structured training interventions have been shown to sustain communication gains and improve physician comfort with sensitive topics [62]. By addressing both medical and non-medical aspects of care, including costs and insurance, the QPL serves as a comprehensive tool for improving satisfaction and patient-centred engagement. Our findings contribute to the growing body of evidence that QPLs enhance communication quality, patient empowerment, and shared decision-making [34,54,60,46,64]. To sustain these benefits, future initiatives could explore digital QPL formats for continuous engagement, incorporate structured financial counselling components, and implement periodic follow-up reinforcement [64].

### Strengths and limitations

To the best of our knowledge, this is the first study to evaluate the effectiveness of a QPL among a tribal population in India. This represents an important step toward culturally grounded, patient-centred communication in an underrepresented region. Despite the positive findings, our study has certain limitations. The analysis focuses on a single cancer centre, which limits generalisability, and the relatively short follow-up period precludes assessment of long-term outcomes. Moreover, consultation duration and qualitative physician feedback were not documented, which could have provided deeper insights into implementation challenges. Future studies should adopt longitudinal, mixed-method designs to assess sustained behavioural change among both patients and providers and explore the feasibility of digital and community-based QPLs tailored for culturally diverse populations.

## Conclusion

In conclusion, our findings highlight the substantial impact of QPLs on enhancing patient satisfaction and engagement during oncology consultations. The QPL empowered patients to participate actively, ask meaningful questions, and address complex topics such as treatment plans and financial concerns by providing a structured framework for discussion. These benefits are consistent with previous studies, demonstrating the versatility and effectiveness of QPLs across diverse healthcare settings. However, sustaining these benefits over time remains a challenge, highlighting the need for complementary interventions such as physician training, financial counselling, and periodic reinforcement.

Integrating QPLs into routine oncology practice and incorporating them into national cancer care protocols, such as those under India’s National Cancer Control Programme, could play a pivotal role in standardising patient–physician communication, reducing anxiety, and fostering patient-centred, informed decision-making across cancer care settings.

## Supporting information

S1 File Annexure 1: Question Prompt List (QPL). (DOCX)

## Abbreviations

QPL: Question Prompt List
WHO: World Health Organisation.
