# Supplementary material for: The effectiveness of Question Prompt Lists (QPL) in enhancing treatment outcomes for cancer patients among tribal population in Meghalaya: A quasi-experimental design
Source: PLoS One. 2025 Dec 22;20(12):e0338482. doi: 10.1371/journal.pone.0338482 (PMC12721520; doi:10.1371/journal.pone.0338482)
Supplement: S1 File — (DOCX) [file pone.0338482.s001.docx]

**Supplementary material**

**Annexure 1: Question Prompt List (QPL)**

**Question Prompt List.**

If you have been told that you have cancer, you may have a lot of questions but are not sure how to ask them. Your healthcare team is the best source of information about your situation.

We advise you to use this list of questions in consultation to help you get the information you need from your doctor. Most people undergoing treatment for cancer have questions and concerns. Still, these are often forgotten in the rush of the moment or because of the stressful environment that can accompany the consultation. Before the consultation, read the brochure and identify the questions you want your doctor to answer or add questions in the space provided. Your doctor will be happy to try to answer any questions you may have. You can use this brochure list during this visit, as you may choose to use it later, or you can discuss these questions with another healthcare team member (e.g., a nurse). Do not feel obligated to ask any of these questions just because they are listed. This booklet lists many questions so that each patient can find some that are appropriate for him or her. For you personally, some of these questions are not relevant to you, so do not stop there. You will likely have other questions as well.

| **Domains** | **Questions** |
| --- | --- |
| 1. When you’re told you have cancer | 1. What kind of cancer do you think I have? 2. Why do you think I have cancer? 3. Where do you think the cancer started in my body? Has it spread from where it started? 4. Do you know the stages of cancer? Would you explain what the stage means for me? 5. What are my chances of surviving cancer? 6. Do I need to have another test? 7. Who will do these tests? When and where will they be done? Who can explain them to me? 8. What are the treatment options available for me? 9. How and when will I get these results? Who will explain them to me? |
| 1. Deciding on a treatment plan | 1. Should I get a second opinion? How will I do that? 2. What are my treatment choices? 3. What treatment do you suggest? Why? 4. How soon do I need to start treatment? 5. What will happen if I decide not to get treatment right now? 6. What is the goal of this treatment? Do you think it could cure cancer? 7. Where can I learn more about this treatment and any other choices I might have? 8. Will I need surgery? 9. If I need surgery, will I need other treatment(s) before or after surgery. 10. What will my treatment be like? 11. How long will my treatment last? 12. Where will I go for treatment? 13. Who will do my treatment? 14. Will these treatments have risks? 15. Will I be able to have children after treatment? 16. How will my treatment affect my work, family, and daily activities? 17. What would we do if the treatment doesn’t work, or the cancer comes back? |
| 1. Before and during treatment | 1. What can I do to get ready for treatment? 2. Should I take a family member along with me for treatment 3. Is there anything I can do to help the treatment work better? 4. What side effects could I have from these treatments? 5. What’s the best time to call if I have a question or problem? 6. What should I do if I have any serious problems during the treatment? 7. How will we know if the treatment is working? 8. Will I need a special test such as imaging scans or blood test, and if so, how often? 9. Will I need to take any special precautions while I’m getting treated? 10. Do I need to change what I eat during treatment? 11. What about vitamins or diets that friends tell me about? How will I know if they are safe? 12. What if I need help at home? 13. Can you suggest a mental health professional so I can see if I start to feel anxious, depressed, or distressed? |
| 1. Symptoms or side effects from your cancer or treatment | 1. What can I do to help control my symptoms and side effects? 2. Who can help me control my symptoms and side effects? 3. What signs, symptoms or side effects should I let my health care team know about? Which ones should I tell you about right way? 4. When should I call you? 5. Is there any special care you’d recommend for me? |
| 1. If your treatment is not working or you need to change to a different treatment | 1. Why do you think this treatment isn’t working for me? 2. What are my options now? Are there other treatments that might work for me? 3. What are the chances a new treatment will work? 4. If you don’t have a good treatment option for me is there somewhere I could get a second opinion? 5. What if I decide I don’t want to get more treatment? |
| 1. If you have finished the treatment or are not getting treated | 1. Now the treatment is done, is there any other medicine or therapy I need? How do you know 2. What type of follow-up will I need? 3. How often will I need to have checkups, blood, and imaging test after my treatment ends? 4. How long will it take for my side effects to go away? 5. What problem should I watch for now that my treatment is finished? 6. Who can I talk to if I have trouble coping with the changes in my life? 7. Are there any limits on what I can do? 8. How will I know if the cancer has come back? What should I watch for? 9. What will we do if the cancer comes back? 10. What can I do to try to keep my cancer from coming back? |
| 1. Treatment cost | 1. What is the overall estimated cost of my cancer treatment? 2. Can you provide a breakdown of the costs associated with each phase of treatment (diagnosis, surgery, chemotherapy, radiation, etc.)? 3. Are there alternative treatment options available with different cost implications? 4. Which cancer medications are covered by my government insurance, and are there generic alternatives available? 5. Can you provide information on any assistance programs for obtaining cancer medications at a lower cost? 6. Are there any hidden costs associated with cancer treatment that patients often overlook? 7. Can you provide information on potential unforeseen expenses that may arise during treatment? 8. How might changes in the frequency or duration of my ongoing treatment impact the overall cost? 9. Can we discuss any potential adjustments to the treatment plan to manage costs effectively?      1. Are there potential unexpected costs that may arise during the ongoing treatment, and how can we prepare for them? 2. Can you provide guidance on managing any unforeseen financial challenges that may emerge? |
| 1. Insurance Coverage | 1. What can I do if I don’t have health insurance? 2. Who can help me out with what my insurance covers and what I will have to pay myself? 3. What can I do if I can’t afford the treatment I need? 4. How much of my cancer treatment costs will be covered by my insurance? 5. What specific treatments or services may not be covered by my insurance plan? 6. Are there any restrictions on the number of treatments or types of medications covered? 7. Is there a financial counselor or specialist I can speak with to discuss my individual financial situation and guide me regarding the available support options and create a plan? 8. Can you guide me through the process of applying for smartcard (MHIS) to help cover cancer treatment costs? 9. What services are covered under MHIS (smartcard), and are there any limitations? |
| 1. Out of pocket expenses | 1. What out-of-pocket expenses can I anticipate during my cancer treatment? 2. How can I estimate my potential out-of-pocket costs for the entire course of treatment? 3. Are there any additional tests, medications, or procedures that may incur extra costs? 4. How can we proactively address the long-term financial impact of ongoing cancer treatment? |
| 1. Treatment Schedule and Work Impact: | 1. How might the treatment schedule affect my ability to work 2. Are there any accommodations or support services available to help manage work-related challenges during treatment? |

**Khasi Version**

Lada don ba iathuh ia phi ba phi don ka jingpang cancer phi lan ban don shibun ki jingkylli, hynrei phim long tikna kumno ban kylli ia ki. Ka team jong ka jingkoit jingkhiah ka long kaba biang palat ban long ka tyllong ban ai ia ka jingtip halor ka bynta kaba phi don. Ngi ai jingmut ia phi ban pyndonkam ia kine ki jingkylli harum. Kum shi bynta ban ai ka jingiarap ia phi ban ioh ki jingtip ba tikna ba phi donkam ban ioh na ki doctor jong phi. Bun ki briew kiba dang don hapdeng ki jingsumar na ka cancer, ki don ki jingkylli kiba iadei bha bad ka jingpang ba ki don. Hynrei, ka jingstet palat ka por la kum klet noh ban kylli, ne namar ka jingpang ba ki don ka pynkulmar jingmut ha ka por ba ki leit ban iakren bad ko doctor. Shwa ban leit jingtip donkam ban pule ia kine ki lyngkdop kot bad wad ia ki jingkylli kiba phi donkam ia u/ka doctor jong phi kin jubab ne phi lah ruh ban buh ia ki jingkylli ha katei ka jaka kaba lait ba la buh na ka bynta kane. U/Ka doctor jong phi un pyrshang da ka jingkmen ban jubab ia ki jingkylli jong phi. Phi lah ban pyndonkam ia ine I kot lyngkdop aijingtip por ba phi leit ia kynduh ia u/ka doctor, ne phi lah ruh ban pyndonkam hadien habud ne lah ruh ban ia mir jingmut bad uno uno u member jong ka health care team (Nuksa ki Nurse).

Wat juh sngewbeij ban kylli ia kine ki jingkylli tang namar ka la buh ia ki. Kane ka kot lyngkdup kadon shibun ki jing kylli, kumta ba uwei pa uwei u nongpang kin shem bad jied kano naki kiba iahap bad ki.

Na ka bynta jong phi shimet, khyndiat na kine ki jingkylli kin yn da iahap palat ia phi wat sngeweh hangtag, phi lah ban don pat da kawei ki jing kylli

| **Ki Kyndon** | **Ki Jingkylli** |
| --- | --- |
| 1. Lada don ba iathuh ba phi pang cancer | - Ka cancer ba kumno ban ga don? - Balei nga don cancer? - Nangno ka bynta jong ka met jong nga kaba kan ka jingpang ka don sdang? Ka la pur ne em naka jak aba ka sdang? - Phi tip katno bynta ka cancer ka don? Phi lah ban batai ka mut aiu kita ki bynta jong ka cancer? - Kiei kita ki lad-lynti ban lait im naka cancer? - Dang donkam ia nga ban leh sa kiwei ki test? - Mano ban leh ia kine ki test? Lano bad hangno kin leh ia kine ki test? Mano ban batai bniah ia nga? - Kumno bad lano nga lah ban ioh ia ki report? Mano ba lah ban pynsngewthuh halor jong kane ia nga? - Lah ne em ban pyndonkam (biomarker or molecular testing) na ka bynta jong kane ka jaid jingpang? - Nga lah ban pyrkhat shaphang ka genetic testing? |
| 1. Ka jingrai halor ka plan jingsumar | - Nga lah ban ioh ia ka jingtip na kiwei pat ki doctor? Kumno ngan leh ia kata? - Kiei ki bynta ba pher ba pher jong ka jingsumar? - Kaba kumno ka lad jingsumar ba phi lah ban ai jingmut? Balei? - Lano donkam ban sdang noh ka jingsumar? - Jia aiu lada ngam kut jingmut ban shim ia ki jingsumar mynta? - Kaei ka jingthmu jong kane ka jingsumar? Phi tharai ba ngan ioh ka jingkoit na kane ka jingpang? - Naei nga hap ban pule shaphang kane ka jingsumar lane da kiwei ki lad ban ga lah ban don? - Hato nga donkam kam puid ne sumar? - Hato lada nga donkam puid , nga donkam ban leh da kiwei pat ki jingsumar hashwa lane hadien ba la dep puid? - Hato don ki clinical trial ba ioadei bad nga? - Donkam ne em ban peit da kiwei pat ki doctor? - Kaba kumno ka jingsumar jong nga kan long? - Haduh katno ka jingsumar jong nga ka dei ban long? - Haei ba nga hap ban shim ia ka jingsumar? - Mano ban leh ia ki jingsumar jong nga? - Hato kine ki jingsumar jong nga ki don ka jingma? - Hato nga lah ban ioh khun hadien kine ki jingsumar? - Kumno ka jingsumar jong nga ka ktah ia ka kam ka jam, kiba ha iing ha sem , bad ia ka jingtrei jong nga? - Ngan leh kumno lada ka jingsumar jong nga kam treikam, lane lada ka cancer jong nga kan wan biang? - Hato ka dang lah ban long ne em ba ngan ioh ia kane ka jingpang hadien ba la dep ioh ka jingsumar? - Mano ban iarap ia nga ha kaba iadei bad ka insurance jong nga bad hato nga hap ban siew dalade? - Ngan leh kumno lada ngam don ka health insurance? - Ngan leh kumno lada ngam lah ban siew ia ka jingsumar ban nga donkam? - Katno ei ei ba nga hap ban lut lada nga shim kane ka jingsumar? |
| 1. Hashwa bad ka por ba sumar | - Kaei ba nga dei ban leh ban pynkhreh ia ka jingsumar? - Nga lah ne em ban iah kali hi, lane nga donkam ban phah wan buh ne shaw? Ngan leh kumno lada ngam don ban wan buh ban shim jingsumar? - Hato nga don ne em ba ngan leh khnang ba ka jingsumar jong nga kan iaid khambha? - Kiei ki side effect ban don na kine ki jingsumar? - Hakano ka por ba biang ban call lada nga don ki jingkylli ne jingeh? - Ngan leh kumno lada nga don ki jingshitom ba jur haka por ka jingsumar? - Kumno ngi lah ban tip ba ka jingsumar ka treikam ne iarap? - Hato nga donkam ki test ba kyrpang kum ki imaging scans lane test snam lada donkam katno sien? - Hato nga donkam ban shim ki jinghusiar ba kyrpang haka por ba dang shim ka jingsumar? - Nga donkam ban pynkylla ia ki jingbam ba nga bam haka por jingsumar? - Hato nga lah ban exercise haka por ba dang sumar? Kum kiei ki jingsumar ban ga dei ban leh man ka por? - Kumno ki vitamins bad ki jingbam ba ki paralok ki ong? Kumno ngan tip ba ki long kiba bha? - Kumno ngan leh lada nga donkam jingiarap ha iing? - Phi lah ban ai jingmut ia nga ki nong iarap ha ka bor pyrkhat pyrdain ban ga lah ban ia kynduh lada nga sngewsih, sngeweh ban pyrkhat, sngewtieng lane sngewshem jingeh? |
| 1. Lada phi don ki dak ki shin lane ki side effects na ka jingsumar jong ka jingpang jong phi? | - Kaei ba nga dei ban leh ban adkar ia ki dak ki shin bad ki jingktah ia ka met ka phad? - Mano ba lah ban iarap ban the la kam ia ki dak ki shim bad ia ki jingktah ia ka met ka phad? - Kiei ki dak ki shin lane ki jingktah ia ka met ba nga dei ban pyntip ia ki nongsumar ka koit ka khiah? Kiei ki ban ga dei ban iathuh da ka ba dei? - Ha kano ka por ba nga dei ban call ia phi? - Hato don ki jingsumar ba kyrpang phi lah ban aijingmut ia nga? |
| 1. Lada ka jingsumar jong phi kam treikam lane phi hap ban pynkylla da kawei ka jingsumar | - Balei phi pyrkhat ba ka jingsumar jong nga kam treikam? - What are my options now? Are there other treatments that might work for me? - Kiei ki lad ba ka jingsumar ba. Thymmai kan iarap? - Lada phim don ki jingsumar ba bha ban jied ia nga , hato dang don shawei shawei ki doctor banga lah ban iakynduh? - Hato kumno lada ngan kwah shuh ban shim ka jingsumar ? |
| 1. Lada phi dep ia ka jingsumar bad phim pat ioh ia ka jingkoit | - Mynta ka jingsumar ka la dep, hato don da kiwei ki dawai lane ki jingsumar ? Kumno ngi lah ban tip - Kiei kita ki follow up ba nga donkam? - Katno sien nga donkam ban phah checkup, test snam, ki test kum ka imaging test hadien ba la kut ka jingsumar? - Katno por phi tharai ba ki jingktah ia ka men (ki side effects) kin kut? - Kiei ki jinggeh ban ga hap ban peit mynta da la kut ka jingsumar?? - Iano nga dei ban kren lada nga don jingeh ban iaishah ha kaba iadei bad ki jingkylla ha ka met jong nga? - Hato don ka limit ban ga lah ban leh? - Kumno ngan tip lada kane ka jingpang kan wan biang? Ia kaei ba nga dei ban peit? - Kumno ngan leh lada kane ka jingpang kan wan biang? - Kiei ki lad ba ngan pyrshang khnang ba kane ka jingpang kan ym wan shuh? |
| 1. Ka jinglut hakaba iadei bad ka jingsumar | - Katno ka jinglut baroh ha kaba iadei bad ka jingsuman jong nga? - Hato long ne em ba phin ai ia ki jingkhein jong ki dor ba pher kiba iadei bad ki bynta jong ka jingsumar?( diagnosis, surgery, chemotherapy, radiation, etc.)? - Ki don ne em da ki wei pat ki lad ki bynta kiba iadei bad ka jingsumar ha ki dor kiba bapher? - Kiei ki dawai cancer kiba lah don ne peit bynta da ka insurance sorkar bad ki don shuh ne em kiwei de kil bynta? - Hato phi lah ban ai jingtip lem ia ki prokram kiba iader ban pyn ioh iaki dawai cancer ha ka dor kaba kham tad lane ba kham biang? - Hato ki don ne em ki dor ki bym tip shai kiba iadei bad ka jingsumar kaba ki briew ki khmih bha? - Hato phi lah ban ai jingtip ia ki jait jait dor ne jinglut kiba lah ban mih hapor ka lingsumar? - Hato ki bynta jong ka jingsumar bad ka jing iaid pateng jong ka jingsumar jong nga kan ktah ia ka jinglut jingsep? - Long ne em ban ngin iakren shphang ka jinsumar ban/ pynbei bad pyniaid bha a ki jingiut jingsep? - Hato kin don ne em ki jinglut jinsep kiba lah ban mih shuh hapor ka jingsumar bad kumno ngin pynkhreh iaki? - Phi lah ne em ban pynbatai lane ai jingmut lem ia kino kino ki jinglut jingsep kiba lah ban mih ha kano kano ka por? |
| 1. Ka jingiarap jong ka insurance | - Ngan Ieh kumno lada ngam don ka Health Insurance Lane Smart Card? - Mano ba iah ban iarap ia nga ha kaba iadei bad kal insurance, hato nga hap ban siew da lade? - Ngan leh kumno lada ngam iakot bor ia ka jingsumar tan ga donkam? - Katno eiei ba ka jingsumar jong nga ka lah ban iarapna ka insurance jong nga/lane smartcard? - Kum kiếi ki kynja jingsumar ki bym lah ban iarap na ka insurance lane na ka smart card jong nga? Hato don ne cnuki jingpyrkhing ha ka ba iadei bad ki jait jingsumar lane ki jait dawai ki ba lah shem bynta na ka insurance? - Hato don ki counsellor na ka liang jong ka jinglut jinsep lane ki nongiarap ban ga lah ban iakren ha kaba iadei bad ka jinglut? - • Phi lah ne em ban batai ia ki rukom kumno ban ioh kil lad jingiarap ia ka jinglut jingsep lyngba ka smart card (MHIS) ban ioh jingsumar ia ka jingpang bampong? - Kiei ki jingiarap kiba lah ban pynlut lyngba ka smart card bad ka don kyndon ne em lane ka kut ne em? |
| 1. Ki jinglut jingsep jong ka jingsumar | • Kaei ka jinglut kaba ngi hap ban pynlut na lade ha ka por suman ia ka jingpang bampong?  • Kumno ngin lah ban buh jingkhein ia ki jinglut jingsep na lade na ka bynta ka jingsumar kaba pura?  • Hato ki don kiwei pat ki test, dawai lane kiwei kiwei ki lad jingsumar pat ba hap donkam ban siew ne ban pynlut na lade?  • Kumno ngin lah ban tehlakam ia ka jingktah ha ka jingioh jingkot ha ka por ba sumar ia kane ka jingpang? |
| 1. Ka Por jong ka jingsumar bad ka jingtyngkhuhkam | - Haduh katno ka por ka ai jingsumar kan ktah ia ka port rei jong nga? - Hato don ne em ki jaka sah lane ki lad ai jingiarap ba lah ban iarap ban pyniaid ne pyntrei ia ki kam jong nga ha ka por ban nga dang shah sumar? |
